# The system-level organisational design framework: a neuro-cognitive trait interaction approach to workplace inclusion for autistic people

**DOI:** 10.3389/fpsyg.2026.1826284

**Published:** 2026-06-17

**Authors:** Jessica Dark

**Affiliations:** Birkbeck, University of London, London, United Kingdom

**Keywords:** autism, neuro-cognitive trait interaction, neurodiversity, organisational design, organisational psychology, sustainable HRM, workplace inclusion

## Abstract

Employment outcomes for autistic people in the United Kingdom (UK) remain persistently low despite increasing attention to workplace inclusion. Drawing on my positionality as an autistic doctoral student in Organisational Psychology, this perspective article examines how workplace inclusion is often approached through individually negotiated adjustments implemented within existing workplace systems and practices. While these approaches provide important forms of support, organisational conditions continue to shape participation in ways that do not fully account for differences in how autistic people think, process information, and communicate. In response, the article introduces the system-level organisational design framework (SLODF), underpinned by a neuro-cognitive trait interaction lens, as a reflexive framework for examining how organisational conditions shape workplace participation across cognitive variation. Drawing on multidisciplinary literature, the article explores how barriers emerge through interactions between neuro-cognitive traits and the organisational domains of environment, systems, people, and policy. Rather than functioning as a predictive or prescriptive model, the SLODF conceptualises workplace inclusion as an ongoing, context-sensitive organisational process. In doing so, the article contributes to broader developments within Organisational Psychology by encouraging reconsideration of workplace inclusion through organisational design and neuro-cognitive trait interaction.

## Introduction

Employment outcomes for autistic people in the United Kingdom (UK) remain persistently low, with only around one third in work (Department for Work and Pensions, 2024). Despite growing attention to workplace inclusion, support for autistic employees is still commonly organised through individually negotiated adjustments [The Equality Act 2010 (UK), 2010]. While such approaches provide important forms of support, they often operate within organisational conditions underpinned by neuro-normative expectations and work environments not always aligned with differences in how autistic people think, process information, or communicate (Erbil et al., 2024; Özbilgin et al., 2025). These tensions highlight the need for approaches to workplace inclusion that prioritise functionality, wellbeing, and sustainable participation within organisational contexts (Zaks, 2024; Rollnik-Sadowska and Grabińska, 2024; Efeoğlu and Kılınçarslan, 2024).

The development of the SLODF emerged through tensions that extended beyond the original focus of my doctoral research. My doctoral journey began by exploring autistic women’s experiences of diagnosis disclosure in the workplace. However, as the study progressed, it became increasingly clear that these experiences were shaped not only by disclosure itself, but also by the wider conditions through which autistic people are studied, understood, and supported. At the same time, engagement with inclusive research methods within Organisational Psychology highlighted tensions within the field, in which dominant definitions of rigour often prioritise consistency, replicability, and generalisability (Islam and Sanderson, 2021; Chirkov, 2024) over responsiveness to cognitive variation. Questions of workplace inclusion, therefore, became increasingly connected to broader concerns about whose knowledge is recognised, legitimised, and acted upon within research and professional practice (Fricker, 2007). These assumptions also shape how autistic people are understood relationally within workplace and research contexts (Thorpe et al., 2024). Collectively, these experiences shifted my focus from understanding autistic people’s experiences of work towards exploring inclusive research and organisational design.

This perspective article draws on my positionality as an autistic doctoral student in Organisational Psychology and is grounded in the neurodiversity paradigm, whereby cognitive variation is understood as part of human diversity (Botha et al., 2024), whilst also recognising the impact of discrimination and structural ableism, whereby organisational and social systems privilege dominant assumptions of functioning, creating barriers for disabled and neurodivergent people (Walker, 2021; Lundberg and Chen, 2024). From this position, I introduce the system-level organisational design framework (SLODF), underpinned by a neuro-cognitive trait interaction lens (Dark, 2023; Dark, 2025), as a reflexive framework for examining how workplace inclusion can be strengthened through ongoing organisational evaluation and design.

Autism has been conceptualised in multiple ways across research, practice, and community contexts, including as a diagnostic category, neurotype, disability, and identity. However, diagnostic interpretations of autism, such as those reflected within the DSM–5–TR (American Psychiatric Association, 2022), continue to shape legislation, workplace support, and broader understandings of autism from a predominantly medicalised perspective. Within this framing, organisational responses to disability often focus on identifying and accommodating individual differences in relation to perceived workplace norms, rather than examining how workplace systems themselves are structured. These approaches emerge from a longer history of framing cognitive differences through treatment and normalisation-oriented practices (Silberman, 2015; Zaks, 2024). Consequently, workplace practices often remain oriented towards normative expectations surrounding communication, behavior, and participation in work (Kapp et al., 2019; Erbil et al., 2024).

These assumptions influence how support is accessed and experienced within organisational settings. Although organisations hold legislative responsibility for providing reasonable adjustments, implementation remains a challenge (Ma, 2023). Access often depends on disclosure of diagnosis, self-advocacy, and the ability to navigate organisational systems not designed for cognitive variation (Nimante et al., 2023). As a result, autistic employees frequently experience pressure to determine whether, when, and how to disclose their diagnosis (Davies et al., 2022; Romualdez et al., 2021). Support, therefore, remains uneven, shaped not only by formal policy but also by how autism, disability, and difference are interpreted across workplace contexts (Davies et al., 2025; Raymaker et al., 2022).

Collectively, these arguments highlight the need for organisations to embed accessibility within the design of work and organisational systems alongside personalised adjustments where required. For the remainder of this article, I present the SLODF as a reflexive approach to workplace inclusion and organisational design that advances three connected shifts in perspective: from diagnosis-led categorisation to neuro-cognitive trait interaction, from individual responsibility to organisational accountability, and from adjustment-led approaches to inclusive organisational design.

## The system-level organisational design framework (SLODF)

The system-level organisational design framework (SLODF) is presented here as a systems-oriented approach to workplace inclusion that positions organisational context and design as central to accessibility, participation, and well-being across cognitive variation (Cooke, 2025; Dark, 2024; Bottema-Beutel, 2024). The framework responds to literature highlighting the need to address environmental and structural barriers within workplace contexts (Manning et al., 2023; Nishith et al., 2025; Kalmanovich-Cohen and Stanton, 2025), alongside autistic community calls for research to inform wider organisational change (Davies et al., 2024).

As a perspective article, literature was selected purposively to support conceptual development of the framework across Organisational Psychology, sustainable HRM, neurodiversity, autism, and disability research, prioritising sources relevant to workplace participation, organisational systems, and inclusive design, alongside socio-ecological and systems-oriented approaches (Özbilgin et al., 2025; Donald et al., 2025; Tomczak and Kulikowski, 2023). Rather than functioning as a predictive or standardised model, the SLODF is intended as a reflexive heuristic for examining how organisational conditions shape workplace engagement across cognitive variation and work settings. Applying a neuro-cognitive trait interaction lens (Dark, 2023; Dark, 2025), the framework examines how sensory processing, attention, and social communication interact with workplace environments, systems, people, and policy to shape participation within organisations.

As shown in Table 1, each domain is explored through four stages: (1) identifying the function of each domain, (2) examining points of interaction between workplace demands and neuro-cognitive traits, (3) identifying barriers and sources of friction emerging through these dynamics, and (4) considering organisational responses that reduce barriers and support participation. Together, these stages provide a structured process for examining how workplace barriers emerge and where system-level redesign opportunities may be most effective.

**Table 1:** Applying a neuro-cognitive trait interaction approach within the SLODF.

Domain	Function (1)	Interaction (2)	Barriers (3)	Design responses (4)
Environment	The physical and sensory aspects of work	Sensory processing × environmental demands	High sensory load affecting participation and sustained engagement	Reduce sensory load through adjustments to lighting, noise, and layout; provide low-stimulation or recovery spaces
Systems	Organisational processes and workflows	Attention × system demands and task structure	Increased cognitive effort may affect task initiation and transitions	Increase clarity and visibility of processes; structure tasks and expectations
People	Communication, relationships, and culture	Social communication × implicit relational norms	Indirect communication and mismatch in communication styles; uncertainty in interpreting intent and expectations	Use clear and direct communication; provide multiple communication formats; implement regular check-ins
Policy	Governance and enactment of inclusion	Policy × organisational implementation practices	Inconsistent implementation of inclusive practices	Ensure consistent and transparent implementation; provide equitable access to support

The following sections present each domain in turn, drawing on research findings to explore how interactions between neuro-cognitive traits and organisational conditions contribute to barriers to participation, and how these barriers may be minimised or overcome through inclusive organisational design.

### Environment

Workplace participation is shaped, in part, by the sensory and physical conditions of the work environment, making environmental design central to accessibility and sustained engagement (Dark, 2023). Many autistic people experience variation in the processing of stimuli such as light, sound, touch, and smell (de Vries, 2021). Consequently, environmental features including open-plan offices, background noise, and fluctuating sensory conditions may interact with differences in sensory processing to influence how work is experienced (Tomczak et al., 2022). Where sensory demands become difficult to manage, particularly within high-stimulation environments, cognitive strain may increase and sustained engagement in work may become more difficult (Dark, 2023; de Vries, 2021).

These demands are not experienced uniformly across organisational contexts. Remote work, for example, may provide greater control over environmental conditions and reduce exposure to high-stimulation settings (Tomczak et al., 2022). At the same time, transitions between environments, routines, and modes of working may introduce ongoing planning and adjustment demands (Pfeiffer et al., 2024; Dark and Lauder, 2025). Over time, repeated adaptation to changing sensory and organisational conditions may contribute to fatigue, overwhelm, and difficulties sustaining engagement in work, with negative repercussions for well-being (van den Boogert et al., 2022; Morris et al., 2025). As such, sensory demands are not solely individual experiences but emerge through interactions between cognitive variation and workplace environments.

Consequently, reducing unnecessary sensory impact becomes an important consideration within organisational design. Approaches may include managing lighting, noise, and temperature, as well as providing access to natural light or low-stimulation spaces (Tomczak et al., 2022; Waisman-Nitzan et al., 2021). Such features can be understood as shared organisational resources that support broader accessibility rather than solely as individually negotiated adjustments. However, no single work environment is universally supportive. Homeworking may reduce sensory intensity while simultaneously introducing challenges relating to informal learning, isolation, or blurred work–life boundaries (Tomczak et al., 2022). These dynamics highlight the importance of flexibility over standardisation within organisational design (Brooks et al., 2024). Research on sensory-inclusive environments further suggests that accessibility is shaped not only by sensory intensity but also by predictability, spatial layout, social understanding, and opportunities for recovery (Manning et al., 2023). From a systems-oriented perspective, designing work environments that respond to sensory variation may support more consistent and meaningful participation across the workforce while shifting responsibility for inclusion from individual adaptation towards the design of workplace conditions.

### Systems

Workplace systems shape how work is understood, navigated, and enacted through information flow, task sequencing, and the visibility of expectations across processes such as recruitment, onboarding, and performance management. Because these processes are embedded within everyday organisational practice, barriers may remain implicit and less visible within organisational decision-making (Dark, 2023). From a neuro-cognitive trait interaction perspective, these system-level conditions interact with cognitive variation, particularly attentional patterns associated with monotropism and inertia (Murray et al., 2005; Lawson, 2025). As such, attention may become strongly focused on specific tasks or interests (Brosnan and Camilleri, 2025). While this may support sustained focus and immersive engagement with tasks of interest, shifting attention between tasks and activities may require greater cognitive effort (Rapaport et al., 2024). Consequently, organisational processes that lack clarity, consistency, or visibility may increase the cognitive effort required to navigate competing work demands and task transitions (Dark and Lauder, 2025).

These dynamics can be observed across the employment lifecycle. Recruitment practices, for example, frequently rely on unstructured interaction and social performance, potentially disadvantaging people whose communication styles differ from dominant workplace norms (Davies et al., 2023). In contrast, more structured approaches, including transparent recruitment stages, clearly sequenced workflows, and role-aligned assessments, may improve clarity and consistency across organisational processes (Nishith et al., 2025; Dreaver et al., 2020; Diener et al., 2020). Similar patterns may extend beyond recruitment, where workplace systems that do not account for cognitive variation may unintentionally reproduce barriers by reducing predictability, accessibility, and clarity within work (Dark and Lauder, 2025). Consequently, workplace inclusion requires consideration of how organisational systems are structured and where to embed supportive expectations, processes, and guidance within workplace design. This shifts responsibility away from relying primarily on individual adaptation to existing systems.

### People

Organisational communication, relationships, and workplace culture shape how participation, collaboration, and understanding occur within work settings. In many workplaces, interaction is guided by implicit workgroup norms and socially expected practices that influence how communication and expectations are expressed, interpreted, and responded to (Longmire et al., 2024). Because these norms are often embedded within organisational culture, differences in communication style may not be immediately recognised, yet they can still significantly influence how people engage with work and with one another (Dark, 2023). From this perspective, variation in social communication becomes an important consideration within workplace inclusion rather than an issue located solely within the individual.

The double empathy problem further highlights that communication differences between autistic and non-autistic people are reciprocal, emerging through mismatches in how meaning is expressed and understood (Milton, 2012; Crompton et al., 2020; Davis and Crompton, 2021). Consequently, workplace communication that relies heavily on indirect or implied meaning may require additional interpretive effort, increasing uncertainty within interaction and collaboration (Longmire et al., 2024; Dark and Lauder, 2025). These demands are often intensified within informal workplace interactions that rely on spontaneity, inference, and shared assumptions, whereas written and structured communication formats may provide greater clarity and additional processing time (Nishith et al., 2025).

Leadership approaches and team cultures further influence how these communication dynamics are interpreted and navigated, particularly where expectations remain implicit or communication relies on imprecise conventions (Longmire et al., 2024). In such contexts, autistic employees may experience pressure to adapt to dominant communication norms or conceal differences, with implications for well-being and ongoing engagement in work (Pryke-Hobbes et al., 2023). From an organisational design perspective, increasing clarity within communication and relational practices may therefore support more accessible workplace interaction. This may be supported through clearer instructions, predictable guidance, direct feedback, and the use of multiple communication formats (Waisman-Nitzan et al., 2019; Diener et al., 2020; Raymaker et al., 2022). Regular check-ins may further clarify expectations (Buckley et al., 2021), while relational supports, such as job coaching, can, in some contexts, facilitate communication between employees and managers (Martin et al., 2022).

Importantly, such practices may support not only autistic employees but also the broader workforce, including employees whose first language differs from the dominant workplace language, by strengthening accessibility and clarity within organisational communication. These approaches may also contribute to psychological safety, understood as the confidence to ask questions, seek clarification, and share ideas within workplace contexts (Edmondson, 1999; Dark, 2023). In turn, embedding inclusive communication practices within organisational systems may support more consistent and meaningful participation across diverse communication styles and workplace interactions.

### Policy

Policy influences how inclusion is formalised, enacted, and sustained across organisational contexts. Unlike the other domains, policy operates less as a direct site of interaction and more as the organisational mechanism through which commitments to inclusion are articulated, coordinated, and maintained over time. Through governance structures relating to recruitment, adjustments, and flexible working practices, policy positions inclusion as an ongoing organisational responsibility linked to equity and rights (Hayward et al., 2019). In doing so, it shapes how expectations, processes, and access to support are defined and enacted across organisational settings, influencing the consistency and transparency of inclusion in practice.

Organisational climate, employees’ confidence to discuss their needs, and recognition of strengths in relation to job demands all affect whether policy results in meaningful inclusion (Waisman-Nitzan et al., 2021). As a result, inclusion is shaped not only by formal documentation but also by how organisational systems and relationships operate in practice. Employee-led groups, including Disability Employee Resource Groups, may further help employees navigate and influence policy implementation, strengthen access to organisational responses, and enable collective advocacy (Davies et al., 2022). Through these processes, policy becomes embedded within workplace practice, influencing how consistently support is accessed, interpreted, and applied across contexts.

From an organisational design perspective, policy can provide the structural conditions that align and sustain accessible environments, clearer systems, and inclusive communication practices over time. Embedding these expectations within governance processes may strengthen accountability and reduce reliance on informal or individually negotiated adjustments. In this way, policy extends beyond compliance to provide a more coordinated, equitable, and sustainable approach to workplace inclusion.

## Discussion

Reliance on individualised workplace adjustments persists due to dominant medicalised framings of autism informing diagnostic, legislative, and organisational interpretations of disability and difference. Because such approaches can often be implemented within existing workplace structures, emphasis on individualised support may reduce incentives to examine or redesign broader organisational conditions. However, the examples illustrated through the SLODF demonstrate how unchanged environmental, systemic, and cultural conditions can continue to reproduce barriers to participation. Consequently, adjustment-led approaches, when unaccompanied by wider organisational change, may require autistic employees to navigate systems not designed for cognitive variation, while support remains inconsistently implemented and misaligned with processing and communication needs (Erbil et al., 2024; Raymaker et al., 2022). In response, the SLODF reframes workplace inclusion as an organisational process emerging through interactions between cognitive variation and organisational conditions, shifting responsibility for inclusion from the individual towards workplace design itself.

This perspective reflects broader neurodiversity principles that recognise cognitive variation as an expected aspect of humanity (Botha et al., 2024) and, therefore, an expected feature of the workforce. Just as workplaces increasingly recognise diversity across gender, age, ethnicity, and religion, differences in how people process information, communicate, and engage with work may also warrant greater recognition within inclusive workplace practice, including obligations relating to disability and reasonable adjustment under The Equality Act 2010 (UK) (2010). In line with system-level considerations, supporting accessibility requires recognising that cognitive variation extends beyond autistic traits to encompass broader forms of neurodivergence, alongside differences associated with stress, ageing, changing life circumstances, and fluctuating work demands. From this perspective, workplace accessibility becomes not only a matter of responding to person-specific support needs but also of organisational design, whereby inclusive systems and practices support a broader range of employees across changing contexts and capacities.

Importantly, this critique is not intended to position organisations as solely responsible for exclusionary practice. Rather, workplace systems, professional norms, and definitions of best practice have developed within wider social contexts shaped by neuro-normative assumptions and structural ableism, whereby organisational and social systems privilege dominant assumptions of functioning and participation, creating barriers for disabled and neurodivergent people (Erbil et al., 2024; Özbilgin et al., 2025; Lundberg and Chen, 2024). From this perspective, Organisational Psychology plays an important role in reconsidering how workplace inclusion and support are understood and enacted across research and practice, particularly through greater reflexivity about the dominant assumptions that shape participation and best practice (Islam and Sanderson, 2021; Chirkov, 2024).

Translating these principles into practice is likely to vary across organisational settings, occupations, and legislative environments. The SLODF is therefore most applicable in workplaces that allow examination and adaptation of organisational systems, structures, and practices. Workforce demographics, occupational demands, and intersectional factors, including age, race, gender, religion, and sexuality, may shape how neuro-cognitive traits are experienced and where redesign is most needed to support accessibility (Crenshaw, 1989; Mallipeddi and VanDaalen, 2022). Responsive organisational design therefore requires attention to the conditions through which participation occurs, drawing on empirical evidence alongside practitioner and neurodivergent insight. Accordingly, inclusive organisational design is best understood not as a universal solution, but as an ongoing organisational process shaped by workforce needs, organisational conditions, and evolving evidence.

Future development of the SLODF requires empirical exploration. Participatory approaches may be particularly valuable in identifying how barriers and points of friction emerge within day-to-day organisational practice (Dark, 2024). Longitudinal and experience-based methods, including diary approaches, could further develop understanding of how inclusion and accessibility shift beyond formal structures or retrospective accounts. Organisational case studies documenting system-level redesign may also help refine the framework by illustrating how workplace systems, practices, and policies are adapted in applied settings. Such work may further explore the extent to which barriers affecting autistic employees also shape broader workforce experiences. Examining both the successful implementation of the SLODF and ongoing challenges may support shared learning across contexts while advancing understanding of how organisational structures influence access to and workplace well-being.

Collectively, these considerations position the SLODF as a reflexive and systems-oriented approach to workplace inclusion that encourages examination of how workplace systems, environments, and expectations shape engagement across work settings. In doing so, the framework contributes to broader discussions within Organisational Psychology regarding how workplace inclusion and support are conceptualised, enacted, and sustained over time. In line with sustainable HRM perspectives centred on the common good (Cooke, 2025), such approaches may further encourage organisations to recognise the broader social, relational, and organisational benefits of designing workplaces that more effectively support cognitive variation. By shifting attention towards organisational design and neuro-cognitive trait interaction, the SLODF may support more accessible, sustainable, and equitable approaches to work for autistic people and the wider workforce.

## Data Availability

The original contributions presented in the study are included in the article/supplementary material, further inquiries can be directed to the corresponding author.
